# Laboratory Diagnosis of Mpox, Central African Republic, 2016–2022

**DOI:** 10.3201/eid2909.230514

**Published:** 2023-09

**Authors:** Sandra Garba-Ouangole, Josephine Bourner, Festus Mbrenga, Ella Gonofio, Benjamin Selekon, Alexandre Manirakiza, Ernest Kalthan, Christian Malaka, Yap Boum, Piero Olliaro, Emmanuel Nakouné

**Affiliations:** Institut Pasteur de Bangui, Bangui, Central African Republic (S. Garba-Ouangole, F. Mbrenga, E. Gonofio, C. Malaka, Y. Boum II, E. Nakouné);; International Severe Acute Respiratory and Emerging Infection Consortium, Pandemic Sciences Institute, University of Oxford, Oxford, UK (J. Bourner);; Ministry of Health and Population, Bangui (B. Selekon, A. Manirakiza, E. Kalthan)

**Keywords:** mpox, viruses, Central African Republic, monkeypox virus

## Abstract

During 2016–2022, PCR testing confirmed 100 mpox cases among 302 suspected cases in the Central African Republic. The highest detection rates were from active lesions (40%) and scabs (36%); cycle thresholds were lower (≈18) than those for blood samples (≈33). Results were consistent for generic primer– and clade I primer–specific PCR tests.

Mpox is caused by the monkeypox virus (MPXV), a double-stranded DNA orthopoxvirus with 2 known clades: clade I (formerly Congo Basin or Central African clade); and clade II (formerly West African clade), which encompasses 2 subclades (IIa and IIb) (*1*–*3*). Cases of mpox have been identified in the Central African Republic (CAR) since 2001 and have increased over time (*4*). The growing number of cases can be explained by the widening geographic spread of the disease and intensified case-finding activities (*5*). However, official figures probably underestimate the incidence of mpox, which principally occurs in remote areas, where many cases may go undetected because of a lack of diagnostic capacity.

The Ministry of Health and Population set up a passive surveillance program for mpox in 2010. Under this program, specimens are collected from all suspected case-patients with illness meeting the standardized case definition (Appendix), which is disseminated to all health professionals in CAR through regular training sessions and posters displayed in health facilities. Specimens are sent for biologic confirmation by PCR to the national reference laboratory at Institut Pasteur de Bangui (IPB). Whenever possible, contact tracing is conducted after identification of confirmed cases.

Since 2016, each specimen received at IPB is tested for MPXV by real-time PCR. After specimen processing, 200 µL of each sample are extracted by using the QIAamp Viral DNA Mini Kit (QIAGEN, https://www.qiagen.com) according to the manufacturer’s instructions. The reactions are performed in 25 µL volume containing 12.5 µL of TaqMan Universal PCR Master Mix (Thermo Fisher Scientific, https://www.thermofisher.com), 4.5 µL of nuclease-free water (Thermo Fisher), 1 µL of each 10 µmol/L primer developed by TaqMan technology (Thermo Fisher), using the generic primer (G2RG) and clade I–specific (C3L) primers and 5 µL of extracted DNA (*6*). On the basis of these same concentrations, varicella zoster virus (VZV) primers (VZV open reading frame 63) are also used (*7*).

## The Study

We conducted a retrospective descriptive study. By using results from all specimens collected from patients with suspected mpox under the national mpox surveillance program during 2016–2022, we aimed to describe the mpox landscape in CAR and evaluate the agreement of mpox test results (including cycle threshold [Ct] values) generated using the G2RG and C3L primers and different specimen types (blood, active lesion, or scab).

**Figure 1:**
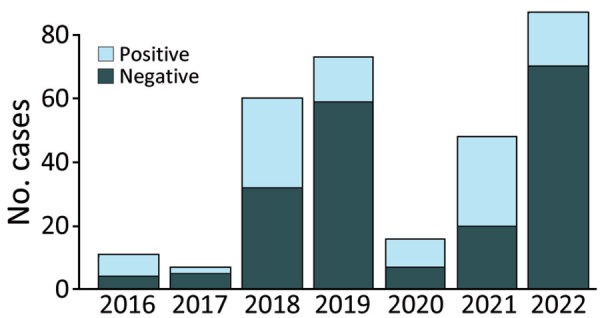
Laboratory test results for persons with suspected mpox cases, by year, Central African Republic, 2016–2022. Of 302 suspected cases during the study period, 105 (35%) had positive results for monkeypox virus on >1 specimen.

During 2016–2022, a total of 494 specimens (278 blood, 99 active lesion, 95 scab, and 22 oropharyngeal) from 302 patients were received and tested for suspected mpox at IPB. Of the total 302 suspected case-patients, 105 (35%) were positive for MPXV on >1 specimen (varying 19%–64% annually) (Figure 1). Of the 105 MPXV-positive patients, 3 (3%) were also positive for VZV. Of the 197 MPVX-negative patients, 82 (42%) were positive for VZV and 108 (55%) were negative for both MPXV and VZV. The remaining 7 patients were not tested for VZV.

The highest percentage of MPXV-positive specimens derived from the Lobaye and Mbomou prefectures, which together contributed 58% of mpox cases overall. MPXV detection rates varied by prefecture: Sangha Mbaere, 24/40 specimens (60%); Lobaye, 35/106 specimens (33%); Mbomou, 25/74 specimens (34%); and Bangui 2/41 specimens (5%) (Appendix Table 1).

Significantly more female patients were among MPXV-positive than VZV-positive case-patients (p = 0.03) but not among case-patients who were negative for both viruses. The median age across all suspected case-patients was 14 years; we observed no statistically significant difference between the median ages of confirmed case-patients with mpox (17 years) and VZV (20 years) infections. The median age of case-patients who tested negative on both tests was significantly lower (9 years) (Appendix Table 1).

Blood specimens were positive for MPXV on G2RG in 77/278 (28%) of cases, active lesions in 45/102 (44%), scabs in 36/98 (37%), and oropharyngeal specimens in 3/22 (14%) (Table 1). Of specimens returning a positive result on G2RG, the median Ct was 32.11 (interquartile range [IQR] 29.12–35.45) for blood specimens, 18.92 (IQR 17.42–23.43) for active lesions, 18.07 (16.19–19.82) for scabs, and 30.15 (28.04–32.56) for oropharyngeal specimens (Table 2). Similar values were returned by C3L. For paired specimens (Appendix Table 2), we observed either substantial (κ 0.61–0.80) or almost perfect (κ 0.81–1.00) agreement of a positive or negative result on pairwise comparisons of tests conducted on different specimens types on either G2RG or C3L.

**Table 1:** Test results by specimen type and test type for MPXV and VZV in a study assessing laboratory diagnosis of mpox, Central African Republic, 2016–2022*

Specimen type	MPXV (G2RG)			MPXV (C3L)			VZV	
	Positive	Negative		Positive	Negative		Positive	Negative
Blood	77/278 (28)	201/278 (72)		73/278 (26)	205/278 (74)		62/260 (24)	198/260 (76)
Active lesion	45/102 (44)	57/102 (56)		45/102 (44)	57/102 (56)		42/108 (39)	66/108 (61)
Scab	36/98 (37)	62/98 (63)		37/98 (38)	61/98 (62)		38/100 (38)	62/100 (62)
Oropharyngeal	3/22 (14)	19/22 (86)		2/22 (9)	20/22 (91)		6/22 (27)	16/22 (73)

*Data are no. positive/no. tested (%). C3L, clade I–specific primer; G2RG, generic primer; MPXV, monkeypox virus; VZV, varicella–zoster virus.

**Table 2:** Cycle threshold values obtained using G2RG and C3L PCR primers on different specimens in a study assessing laboratory diagnosis of mpox, Central African Republic, 2016–2022*

Specimen type	MPXV (G2RG)	MPXV (C3L)	VZV
Blood	32.11 (29.12–35.45)	32.93 (30.25–35.94)	34.41 (31.38–36.01)
Active lesion	18.92 (17.42–23.43)	19.61 (18.05–23.57)	19.23 (17.69–20.82)
Scab	18.07 (16.19–19.82)	18.13 (16.46–21.46)	15.78 (13.63–18.42)
Oropharyngeal	30.15 (28.04–32.56)	28.19 (26.79–29.59)	34.31 (32.95–35.67)

*Data are median cycle threshold value (interquartile range). C3L, clade I–specific primer; G2RG, generic primer; MPXV, monkeypox virus; VZV, varicella–zoster virus.

**Figure 2:**
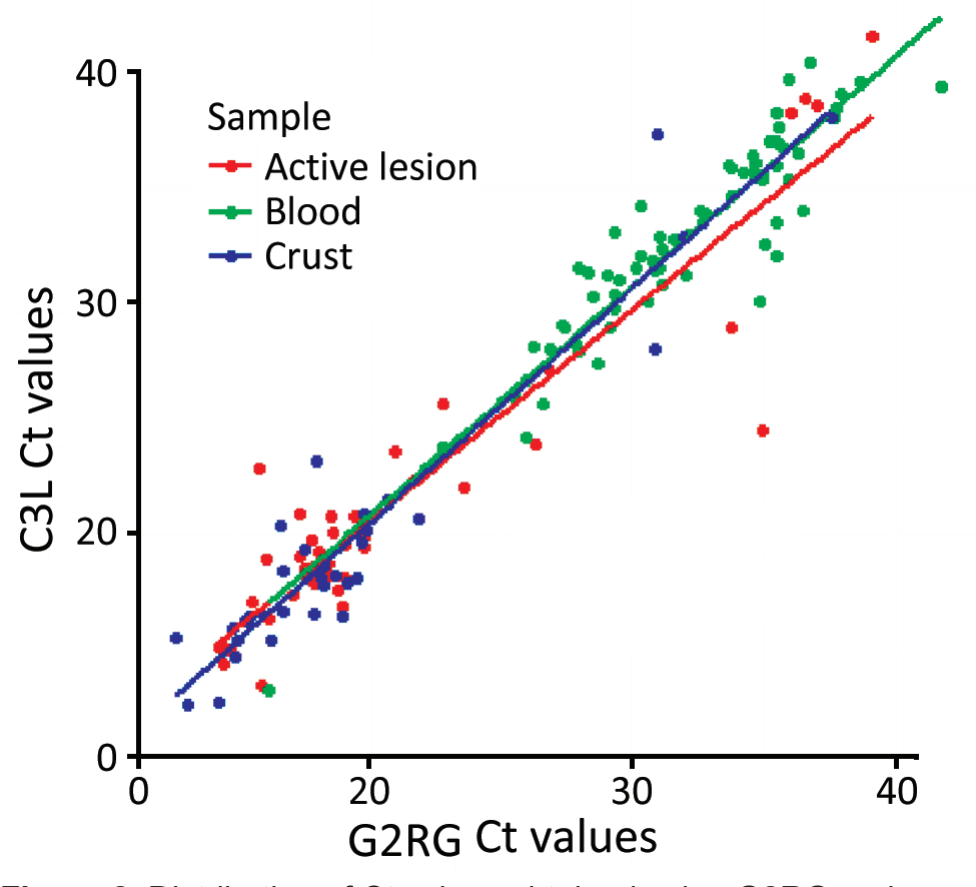
Distribution of Ct values obtained using G2RG and C3L primers of monkeypox virus–positive active lesion, blood, and scab specimens in study assessing laboratory diagnosis of mpox, Central African Republic, 2016–2022. C3L, clade I–specific primer; Ct, cycle threshold; G2RG, generic primer.

The Ct values of G2RG and C3L on blood were significantly higher than in active lesion and scabs, whereas we observed no difference between active lesion and scab specimens. We observed no statistically significant difference between the Ct values generated on G2RG and C3L on the same specimens. (Figure 2)

## Conclusions

Approximately one third of suspected mpox cases in CAR are confirmed MPXV infections; an additional 2/5 are VZV infections, leaving ≈3/5 cases of papulovesicular cutaneous eruptions undiagnosed. Most mpox and VZV infections were diagnosed in teenagers and young adults, with an even younger population remaining undiagnosed.

Although cases of mpox are generally detected across the heavily forested, southern parts of CAR, mpox detection rates vary across prefectures. Some prefectures, such as Sangha Mbaere, have a high detection rate of MPXV (60%) over VZV (5%), whereas in others, such as Bangui, detection is much lower (MPXV 5%, VZV 46%). The varying detection rates between prefectures could be linked to local lifestyles and practices, as well as social instability. In the southwest region, local communities primarily subsist through hunting and gathering, spending long periods in mpox-endemic forest, which may increase the risk for exposure to the virus; however, in the southeast, mpox-endemic bushlands are used for farming and as a place of passage or temporary habitation for communities that have been displaced by social instability.

Our study also detected significantly more female patients among mpox-positive than VZV-positive cases, which may be explained by increased risk for infection through multiple routes of exposure to potentially infected sources. For example, women are primarily responsible for skinning and cooking wild game hunted in the forest and are the primary caretakers for family members who fall ill.

Our results demonstrate very high agreement in PCR results between primers. The results also highlight the need to prioritize active lesion and scab specimens over blood specimens, given that their relatively higher viral loads for MPXV and VZV enable better detection.

CAR faces special geographic, social, and healthcare challenges, leading to substantial delays between symptoms onset, diagnosis, and care. The reported case-fatality ratio for clade I mpox cases varies widely and is often cited as 11% (*8*) but has also been as low as 1.4% in Democratic Republic of Congo (P.R. Pittman et al., unpub. data, https://doi.org/10.1101/2022.05.26.22273379) and 6.7% in CAR (*9*). To improve patient outcomes in CAR, diagnostic capacity needs to be strengthened through greater availability of point-of-care testing and through support by more active epidemiologic and genomic surveillance that can be implemented with a wider range of partners.

Appendix Additional information about laboratory diagnosis of mpox, Central African Republic, 2016–2022.
