# Supplementary material for: Laboratory Diagnosis of Mpox, Central African Republic, 2016–2022
Source: Emerg Infect Dis. 2023 Sep;29(9):1846–9. doi: 10.3201/eid2909.230514 (PMC10461668; doi:10.3201/eid2909.230514)
Supplement: Appendix — Additional information about laboratory diagnosis of mpox, Central African Republic, 2016–2022. [file 23-0514-Techapp-s1.pdf]

Article DOI: https://doi.org/10.3201/eid2909.230514

*EID cannot ensure accessibility for supplementary materials supplied by authors. Readers who have difficulty accessing supplementary content should contact the authors for assistance.*

# Laboratory Diagnosis of Mpox, Central African Republic, 2016–2022

## Appendix

## Case Definitions for Suspected and Confirmed Mpox

The following mpox case definitions are communicated to health professionals across the Central African Republic in training sessions. Flyers containing the case definitions are provided to participants during the training for display at their health facilities in locations visible to anyone who enters.

### Suspected case

Any patient who presents to a health facility with the following signs and symptoms:

- Fever or suspected fever
- Cutaneous rash over the body, particularly on the palms of the hands or soles of the feet
- Adenopathy

### Confirmed case

Any patient whose sample is confirmed by PCR for mpox in the laboratory

## Procedure for Mpox Sample Collection

The standard procedures for collection mpox samples are as follows:

- Written or oral consent is obtained from the patient (or a legal guardian if the patient is a minor)
- Completion of the case notification form provided under the national surveillance program

• Trained healthcare personnel collect the following sample(s):

  o Blood is collected at the onset of the acute phase of the disease or during the late phase after all lesions have desquamated

  o If lesions have not scabbed, a lesion swab is collected

  o If lesion have scabbed, the crusts are removed when the lesions have dried but are still stuck to the body

• All samples are sent to the national reference laboratory at Institut Pasteur de Bangui for testing by PCR using G2R-G and C3L primers

**Appendix Table 1.** Summary of age and sex of patients whose samples were sent to IPB for MPXV testing§

| Characteristic | All | MPXV+ only | VZV+ only | VZV– and MPXV– |
|---|---|---|---|---|
| Sex, ratio F:M | 147:152 | 52:44* | 31:49 | 56:52 |
| Age (years), median (Q1, Q3) [range] | 14 (5,27) [0 to 85] | 17 (6,28) [0 to 67] | 20 (8, 29) [0 to 80] | 9 (2, 21) [0 to 85] |
| Age (years), mean (std) | 18 (14.5) | 19 (13.6) | 21 (15) | 13** (13.9) |
| Prefecture in which sample collected, N (%) | | | | |
| Haut Mbomou | 1 (<1%) | 0 (0%) | 0 (0%) | 1 (1%) |
| Nana Mambere | 2 (<1%) | 0 (0%) | 1 (1%) | 1 (1%) |
| Haute Kotto | 7 (2%) | 3 (3%) | 2 (2%) | 2 (2%) |
| Membere Kadei | 6 (2%) | 3 (3%) | 1 (1%) | 2 (2%) |
| Ombella M'poko | 6 (2%) | 5 (5%) | 1 (1%) | 0 (0%) |
| Ouaka | 16 (5%) | 7 (7%) | 2 (2%) | 7 (6%) |
| Sangha Mbaere | 40 (13%) | 24 (23%) | 2 (1%) | 15 (14%) |
| Bangui | 41 (14%) | 2 (2%) | 20 (24%) | 19 (19%) |
| Mbomou | 74 (25%) | 25 (24%) | 23 (28%) | 23 (22%) |
| Lobaye | 106 (35%) | 35 (33%) | 33 (38%) | 37 (34%) |

§3 cases positive for both MPXV and VZV excluded
*MPXV + vs VZV + chi-square p = 0.03
**MPXV + vs VZV & MPXV- Welch Two Sample t-test = 0.003748; VXV+ vs VZV & MPXV- = 0.0004724
NOTE: MPXV detection rates: Sangha Mbaere = 60%; Lobaye = 33%¸ Mbomou = 34%; Bangui = 5%

**Appendix Table 2.** Comparison of results between blood and scab samples using the G2RG primer

| Comparison | Samples included in pairwise comparisons |
|---|---|
| Blood v. scab (G2RG)* | 82 |
| Blood v. active lesion (G2RG)* | 82 |
| Scab v. active lesion (G2RG)* | 43 |
| G2RG v. C3L (blood)** | 265 |
| G2RG v. C3L (scab)** | 93 |
| G2RG v. C3L (active lesion)** | 95 |

*Comparisons were conducted where the G2RG test was performed on both samples types
**Comparisons were performed where the sample was tested using both primers
